# Mediating Effect of Personal Meaning in the Prediction of Life Satisfaction and Mental Health Problems Based on Coronavirus Suffering

**DOI:** 10.3389/fpsyg.2021.638379

**Published:** 2021-03-16

**Authors:** Gökmen Arslan, Murat Yıldırım, Mega M. Leung

**Affiliations:** ^1^Department of Psychological Counseling and Guidance, Mehmet Akif Ersoy University, Burdur, Turkey; ^2^International Network on Personal Meaning, Toronto, ON, Canada; ^3^Department of Psychology, Ağri İbrahim Çeçen University, Agri, Turkey; ^4^Department of Psychology, University of Leicester, Leicester, United Kingdom; ^5^Independent Researcher, Vancouver, BC, Canada

**Keywords:** suffering, personal meaning, well-being, mental health, COVID-19, second wave positive psychology

## Abstract

**Research Problem:** The onset of the COVID-19 pandemic has triggered a multi-faceted crisis worldwide. Researchers and health authorities in various parts of the world echoed the dire condition of the public's mental health. This study sought to examine the mediating effect of personal meaning on the association between coronavirus (COVID-19)-related suffering, mental health problems, and life satisfaction. Participants included 231 adults (mean age = 46.65 ± 13.98; 68% female) and completed measures of suffering related to COVID-19, meaning, life satisfaction, and mental health problems online.

**Results:** Findings from mediation analysis showed that suffering had significant associations with personal meaning, mental health, and well-being. Furthermore, personal meaning was significantly associated with adults' mental health and well-being and mediated the negative effect of suffering on mental health and well-being.

**Discussion:** Overall, results from this study indicate that personal meaning is an important promotive factor that may help to understand the negative effect of coronavirus-related suffering on mental health and well-being amid the public health crisis.

## Introduction

The initial discovery of the Novel Coronavirus (COVID-19) outbreak in late December 2019 and its subsequent declaration by the World Health Organization (WHO) as a worldwide pandemic on March 11, 2020, had since launched the world into an unprecedented time of uncertainty, threat, grief, and unrest (World Health Organization, 2020). Virus epicenters in various parts of the world struggled to contain the spread of the COVID-19 virus and the death and infection rates continued to soar as the pandemic progressed (Khosrawipour et al., 2020). As of January 19, 2021, the world has recorded 96,052,592 cases of infection and 2,050,580 deaths (Worldometer, 2021). At the time of writing this paper, Canada was undergoing its second wave of the COVID-19 pandemic with a current record of 715,072 cases and 18,120 deaths (Worldometer, 2021). Medical professionals and experts around the world raced against time to develop a vaccine aimed to preclude further harm to human health and additional lives lost. Meanwhile, the public continued to endure the restrictions and health advisories according to their local authorities in the hope to contain the transmission rate and “bend the curve.” Research conducted during pandemic suggests that psychological strengths (e.g., meaning in life, resilience, and hope) function as protective factors against the adverse effects of the pandemic on mental health (Arslan, 2020; August and Dapkewicz, 2020; Burke and Arslan, 2020; Yildirim and Arslan, 2020). Hence, understanding the effect of strengths in the context of the COVID-19 pandemic is important to provide intervention services for fostering mental health and well-being. To this end, the purpose of the present study is to investigate the mediating effect of personal meaning on the association between coronavirus (COVID-19)-related suffering, mental health problems, and life satisfaction.

### Suffering in the Context of the Covid-19 Pandemic

Suffering, according to Frankl (1985), is an inevitable condition of life. A phenomenological conceptualization suggests that suffering is a potentially alienating mood that involves painful experiences at different levels: one's embodiment, engagements in the world with others, and core life values/goals. Furthermore, suffering has the capacity to create a sense of displacement from others and one's own life values and goals (Svenaeus, 2014). Building upon the phenomenological definition, Bueno-Gómez (2017) proposes that a comprehensive definition of suffering should include the existential dimension as it pertains to a person's attitudes, choices, and capacity in dealing with the adverse circumstance. Thus, suffering is defined as a negative experience, which adversely affects an individual at psychological, physical, social, and existential levels.

In the context of the COVID-19 pandemic, research has indicated that individuals' perceived threats of economic, health, security, and political risks; perceived scarcity of available psychological or social resources and personal protective equipment; societal stigma; as well as lack of trust for their government and policymakers' ability to manage the crisis further contribute to one's level of distress (Caqueo-Urízar et al., 2020; Généreux et al., 2020; Shigemura and Kurosawa, 2020). The “new normal” in the pandemic era introduced many new changes to our livelihood, everyday practices, and social behaviors. Many of these changes created confusions and frictions among the social groups such as discrimination and racism (Liu and Modir, 2020; Misra et al., 2020) and contributed to one's anxiety, guilt, loneliness, boredom, anger, and grief (Groarke et al., 2020; Park and Park, 2020). A phenomenon recently surfaced in the media known as “COVID fatigue” describes the psychological exhaustion from the prolonged stress and vigilance of the pandemic. Hence, the new situation has impelled a surge in psychopathy, psychological distress, emotional disturbance, and suicidal risk among the general population (Banerjee and Bhattacharya, 2020; Sher, 2020).

### Mental Health and Well-Being During Pandemic

The current global mental health crisis instigated by the COVID-19 pandemic presents a dire need for the world's mental health and existential well-being. The enforcement of social distancing, isolation, and home confinement measures have further exacerbated the negative impacts on the public's well-being and social health (Ammar et al., 2020; Saltzman et al., 2020; Zhang et al., 2020). Therefore, it is crucial for researchers and clinicians to identify the key factors that help build resilience and propel the mental health and well-being of the public. Chen and Bonanno (2020) suggest that investigation on the long-term patterns of the impact of COVID-19 on mental health is important in order to better understand the multiple risk and resilience factors involved. Such findings can provide valuable information to be incorporated into preventative measures and interventions that help the general public cope with the evolving multi-faceted challenges at different stages of the pandemic (Burke and Arslan, 2020). Researchers have also observed a rise in emotional distress, virus-related distress, panic, and depression among Canadians when social distancing measures were implemented in the early days of the pandemic (Best et al., 2020). A study conducted by Rossi et al. (2020) found that there were relatively high rates of post-traumatic stress, depression, and anxiety symptoms among the Italian population and that these outcomes were associated with COVID-19-related risk factors. Arslan and Yıldırım (2021) reported the significant predictive effect of coronavirus stress on depressive symptoms among Turkish young adults.

Life satisfaction is conceptualized into two broad components: an affective component (i.e., positive affect and negative affect) and a cognitive/judgmental component (i.e., life satisfaction; Diener, 1984, 2017). In this study, we will focus on life satisfaction. Life satisfaction denotes a person's conscious cognitive judgment of one's perceived life circumstance compared with a self-imposed set of standards that defines a good life (Pavot and Diener, 1993; Moore and Diener, 2019). It is expected that, under favorable and predictable conditions, individuals would rate higher on life satisfaction when their perceived life circumstances more or less align with their expectations. It is therefore of interest to determine if individuals' life satisfaction would sustain amid uncertain and adverse conditions such as the current pandemic era. Moreover, the life satisfaction system and the meaning-making processes are complementary to one another. Shmotkin (2005) contends that whereas life satisfaction can moderate the dissonance brought about by the threats from the hostile environment, the process of meaning-making allows one to reconcile with the appraised threats by modifying existing beliefs, values, goals, and assumptions to rebuild a more adaptive world (Park, 2010). Arslan and Allen (2021) reported the significant predictive effect of coronavirus stress on life satisfaction among Turkish young adults. Similarly, Arslan (2021) found that the coronavirus experience had a significant predictive effect on life satisfaction. Researchers highlighted that life satisfaction is not just an outcome or a by-product of other processes, but rather a dynamic system that helps to maintain a positive state of mind by regulating perturbing disruptions from actual or potential threats (Shmotkin and Shrira, 2012; Yildirim and Arslan, 2020).

### Personal Meaning and Coronavirus-Related Suffering

Personal meaning is defined as an individually construed system based on values and can endow life with personal significance and satisfaction (Wong, 1989). Evidence of the positive contributions of meaning to well-being has been well-established in research (Steger and Frazier, 2005; King et al., 2006; Testoni et al., 2018). Moreover, research findings have confirmed the association between the lack of meaning and psychological distress (Steger et al., 2006; Ho et al., 2010). In relation to the second wave of positive psychology (Wong, 2011), personal meaning is pertinent to both suffering and flourishing aspects of life by serving a dual purpose dependent on the individual's circumstance (Wong, 2010). In confronting highly stressful situations, individuals can attempt to alleviate their distress by reconciling the discrepancy between the appraised meaning of the situation and the previously held beliefs/assumptions through meaning-making (Park, 2010). On the contrary, under favorable conditions, meaning can enhance positive affect and enjoyment in mundane everyday living experiences (King and Hicks, 2012) and improve quality of life by engaging in meaningful activities (Iwasaki, 2007). Personal meaning is therefore an important source for well-being and a protective factor against mental health problems and suffering pertinent to this tumultuous time (Arslan and Yildirim, 2020; de Jong et al., 2020; Schnell and Krampe, 2020).

In the context of the COVID-19 pandemic, burgeoning studies support that personal meaning serves as a protective factor against psychological distress amid the viral pandemic (Arslan and Yildirim, 2020; Gori et al., 2020; Yildirim et al., 2020). A renewed sense of personal meaning is suggested to help individuals cope with the traumas and grief over the loss of normalcy during these challenging times (de Jong et al., 2020; Lin, 2020; Schnell and Krampe, 2020). Furthermore, personal meaning has demonstrated to promote life satisfaction, healthy behaviors, and adaptive coping during COVID-19 (Gori et al., 2020; Lin, 2020; Minkkinen et al., 2020). In particular, a person's high meaning in life, life satisfaction, and hope can buffer the psychological stress and fear evoked by the virus pandemic and propel individuals toward altruism in a time that the world needs most (Trzebiński et al., 2020). This call for altruism through personal meaning aligns with the existential transformation process of self-transcendence, which is suggested to overcome suffering and promote well-being (Frankl, 2000; Wong, 2016).

The literature regarding the associations between coronavirus suffering, personal meaning, life satisfaction, and mental health problems mainly focused on the direct link between those variables. The underlying mechanism between the coronavirus suffering and life satisfaction and psychological health problems remains unknown. Therefore, we examined the mediating role of personal meaning in the association between the coronavirus suffering and life satisfaction and psychological health problems. Given the empirical evidence and theoretical framework, personal meaning is key to promote mental health and well-being, which may mediate the adverse impacts of coronavirus experiences on these outcomes. Based on the aforementioned rationale and extant literature, we hypothesized that personal meaning would mediate the relationship between coronavirus suffering and life satisfaction as well as the relationship between coronavirus suffering and mental health problems.

## Methods

### Participants

The participants of this study comprised adults (*n* = 231) residing in Canada and ranged in age between 18 and 83 years (*M* = 46.65; *SD* = 13.98). The study sample is composed of 68% female and 32% male. The majority of adults that participated in the study came from Canada (70.1%), USA (5.6%), and China (3.9%). A web-based survey was created using the study measures and demographic questions. The participants were recruited through social media and the International Network on Personal Meaning's Facebook page to complete the survey on an online platform. Participants were informed of the voluntary nature of the study and were assured that no identifiable personal information was collected to ensure anonymity. Participants were not compensated for their involvement. The study was also approved by the Ağri İbrahim Çeçen University Institutional Review Board.

### Measures

#### Suffering Measure During COVID-19 (SM-COVID-19)

Suffering Measure during COVID-19 (SM-COVID-19) was used to measure individual's coronavirus-related suffering experiences (Wong, 2020). The measure is a 10-item self-report scale (e.g., “Poor physical health condition,” “Poor personal financial condition”); see the appendix for a copy of full list of items. All items were scored using a five-point Likert-type scale, ranging from 1 (not at all) to 5 (a great deal). A total score ranges between 10 and 50, with higher scores indicating greater suffering experiences during the pandemic. Due to the lack of reliability and validity evidence regarding this scale in the available literature, we tested the reliability and validity of the scale using the current sample. Factor structure of the scale was affirmed using confirmatory factor analysis, which indicated an adequate data model fit with the sample of this study –χ^2^ = 83.95, *df* = 32, *p* < 0.001, CFI = 0.94, TLI = 0.91, SRMR = 0.054, RMSEA (95% CI) = 0.084 (0.062, 0.10). The scale had adequate-to-strong factor loadings, ranging between 0.45 and 0.75. In this study, convergent validity evidence was established using bivariate correlations, which showed a satisfactory relationship between the coronavirus suffering and personal meaning, mental health, and life satisfaction. The internal reliability estimate of the scale with the present study is also presented in Table 1.

**Table 1:** Descriptive statistics and correlation results.

		**Descriptive statistics**					**Correlations (*****r*****)**			
**Measure**		***Mean***	***SD***	**Skew**.	**Kurt**.	**α**	**1**.	**2**.	**3**.	**4**.
1.	Suffering during COVID-19	24.23	7.97	0.45	−0.29	0.86	–			
2.	Personal meaning	110.54	17.87	−0.78	1.73	0.89	−0.36	–		
3.	Mental health problems	18.46	5.61	0.72	0.30	0.87	0.60	−0.40	–	
4.	Life satisfaction	23.45	6.08	−0.69	0.27	0.89	−0.41	0.57	−0.49	–

*All correlations are significant at the 0.001 level (two-tailed)*.

#### Personal Meaning Profile (PMP-B)

The brief version of the Personal Meaning Profile (PMP-B) was used to assess individual's perception of personal meaning and sense of purpose and personal significance in their lives (McDonald et al., 2012). The PMP-B is a 21-item self-report measure (e.g., “I believe I can make a difference in the world”). Participants indicate their agreement with each item on a seven-point Likert-type scale, ranging from 1 (not all all) to 7 (a great deal). A total score ranges between 21 and 147, with higher scores indicating greater sense of personal meaning.

#### The Hopkins Symptoms Checklist (HSCL-10)

The Hopkins Symptoms Checklist (HSCL-10) was used to measure individual's psychological distress (Kleppang and Hagquist, 2016). The HSCL-10 is a self-report scale (e.g., “Suddenly scared for no reason”), ranging from 1 (Not at all) to 4 (Extremely). The scale was designed to measure anxiety and depression symptoms and how much these symptoms bothered participants during the last week: with four items about anxiety and six about depression. A total score ranges between 10 and 40, with higher scores indicating greater mental health problems.

#### The Satisfaction With Life Scale (SWLS)

The Satisfaction with Life Scale (SWLS) was used to assess people's cognitive emulations of the life. The scale is a five-item self-report scale (e.g., “I am satisfied with my life”) responding based on a seven-point Likert-type scale, ranging from 1 (strongly disagree) to 7 (strongly agree; Diener et al., 1985). A total score ranges between 7 and 35, with higher scores indicating greater satisfaction with life.

### Data Analysis

Descriptive statistics (e.g., mean, standard deviation) were firstly reported. Normality assumption was checked using kurtosis and skewness scores with their decision rules (skewness and kurtosis scores < |2|; D'Agostino et al., 1990; Field, 2009). Subsequently, correlation analysis was performed to examine the relationship between suffering during COVID-19, personal meaning, mental health, and life satisfaction. Mediation analyses were conducted to investigate the mediating role of personal meaning and life satisfaction in association between suffering during COVID-19 and mental health problems using the PROCESS macro for SPSS version 3.5 (Hayes, 2018). We carried out two independent mediation models (Model 4) to examine the mediating effect of personal meaning on the link of suffering with mental health and well-being. Furthermore, the significance of indirect effects was examined using the bootstrapping method with 10,000 resamples to estimate the 95% confidence intervals (Preacher and Hayes, 2008; Hayes, 2018). All data analyses were performed using SPSS version 25.

## Results

Descriptive statistics and correlation analysis results for the variables in the study are presented in Table 1. Skewness and kurtosis values suggested that all measures in the study had relatively normal distributions, ranging between −0.29 and 1.73. Reliability results also indicated that the internal reliability estimates of the measures with the present sample of the study ranged between 0.86 and 0.89, as shown in Table 1.

Additionally, correlation analysis was used to examine the association between variables of the study. Findings from this analysis showed that suffering during COVID-19 had significant and negative correlations with people's personal meaning and life satisfaction, as well as had a positive association with their mental health problems. Mental health problems were also negatively correlated with life satisfaction and personal meaning. There was a significant and positive relationship between personal meaning and life satisfaction, as seen in Table 1.

We tested the mediating effect of personal meaning in the association between suffering during COVID-19 and life satisfaction and mental health problems. Results from the first mediation model, which was conducted to examine the mediating effect of personal meaning on the link of coronavirus suffering with mental health problems, indicated that suffering had a significant predicative effect on personal meaning (β = −0.36, *p* < 0.001) and mental health problems (β = 0.53, *p* < 0.001). Furthermore, personal meaning significantly mediated the negative effect of coronavirus suffering on people's mental health (β = −0.21, *p* < 0.001). Suffering during COVID-19 accounted for 13% of the variance in personal meaning, and suffering and personal meaning together explained 40% of the variance in the mental health of people, as shown in Table 2. Findings from the second mediating analysis, which was conducted to examine the mediating effect of personal meaning on the link of coronavirus suffering with life satisfaction, revealed that suffering during COVID-19 was significantly associated with life satisfaction (β = −0.23, *p* < 0.001), and personal meaning significantly mediated the effect of suffering during COVID-19 on life satisfaction (β = 0.49, *p* < 0.001). Coronavirus suffering and personal meaning together accounted for 38% of the variance in people's life satisfaction, as shown in Table 2. These results indicate that personal meaning is an important source that can mediate the adverse impacts of coronavirus experience on mental health and well-being.

**Table 2:** Unstandardized coefficients for the mediation model.

	**Consequent**			
	***M*** **(Personal meaning)**			
**Antecedent**	**Coeff**.	***SE***	***t***	***p***
*X* (Coronavirus suffering)	−0.81	0.14	−5.91	<0.001
Constant	130.30	3.52	37.03	<0.001
	*R*^2^ = 0.13			
	*F* = 34.94; *p* < 0.001			
	***Y*** **(Life satisfaction)**			
*X* (Coronavirus suffering)	−0.18	0.04	−4.18	<0.001
*M* (Personal meaning)	0.17	0.02	8.72	<0.001
Constant	9.40	2.68	3.50	<0.001
	*R*^2^ = 0.38			
	*F* = 69.11; *p* < 0.001			
	***Y*** **(Mental health problems)**			
*X* (Coronavirus suffering)	0.37	0.04	8.53	<0.001
*M*(Personal meaning)	−0.06	0.02	−1.37	<0.001
Constant	16.63	2.42	7.79	<0.001
	*R*^2^ = 0.40			
	*F* = 77.13; *p* < 0.001			
**Indirect effect of suffering on mental health**				
	**Effect**	***SE***	**BootLLCI**	**BootULCI**
Suffering → Meaning → Satisfaction	−0.14	0.03	−0.20	−0.07
Suffering → Meaning → Health problems	0.05	0.02	0.02	0.09

*SE, standard error; Coeff., unstandardized coefficient; X, independent variable; M, mediator variables; Y, outcomes or dependent variables. Number of bootstrap samples for percentile bootstrap confidence intervals: 10,000*.

## Discussion

The present study examined the associations between the COVID-19-related suffering, personal meaning, life satisfaction, and mental health in an adult sample. The correlation results indicated that the COVID-19-related suffering and mental health were significantly and negatively correlated with personal meaning and life satisfaction with medium to large effects. These findings demonstrate that as the scores on suffering and mental health increase, the scores on personal meaning and life satisfaction decrease and vice versa. These results are similar to earlier research showing a similar pattern of the emerging relationships among the study variables (McKnight and Kashdan, 2009; Wong, 2019). Intercorrelations between the COVID-19-related suffering, personal meaning, life satisfaction, and mental health revealed in the correlation analysis can be explained by the mediation analysis. The results of mediation analysis indicated significant direct effects of antecedents on consequents. The direct associations between COVID-19 suffering, personal meaning, life satisfaction, and mental health problems were significant. Furthermore, the mediating effect showed an indirect effect of COVID-19 suffering on life satisfaction and mental health problems though personal meaning. Personal meaning mediated the adverse effect of suffering on well-being and mental health. This suggests that the COVID-19 suffering negatively affects the sense of personal meaning, which, in turn, possibly leads to poor life satisfaction and greater mental health problems.

Evidence regarding suffering in the context of COVID-19 pandemic is very limited. In a recent study, Edwards and Van Tongeren (2019) highlighted the mediating effect of meaning in life in the association between suffering and well-being outcomes. In particular, higher suffering was found to be associated with lower meaning in life, which, in turn, reduces satisfaction with life and increases anxiety, depression, and PTSD-related symptoms. According to Frankl (1985), suffering is an inevitable part of life and psychological problems can arise from existential emptiness because of a lack of meaning in life. However, people can find meaning in life from such suffering experiences and challenges. A sense of meaning in life can contribute to mental health of people in difficult times and act as a protective factor against psychological problems (Yildirim et al., 2020, 2021). Suffering weakens a sense of meaning in life, and individuals may experience less life satisfaction and more negative mental health outcomes (Edwards and Van Tongeren, 2019). However, if people can find meaning in the midst of their suffering, they reported greater life satisfaction and less mental health problems. Meaning in life was also related with better psychological adjustment (Park et al., 2008), growth and healing (Wong, 2011; Leung, 2019), lower post-traumatic stress disorders (Updegraff et al., 2008), and psychological distress (Steger et al., 2006; Ho et al., 2010). That is to say, as personal meaning increases, life satisfaction increases, and mental health problems decrease.

The present findings have important implications for future research and practices in terms of mental health services. Although the associations between meaning in life, life satisfaction, and mental health have been examined in the context of COVID-19 pandemic (Yildirim and Arslan, 2020; Yildirim et al., 2020), this study focused on a mediator mechanism that explains the association of suffering during COVID-19 pandemic with life satisfaction and mental health. The current results indicated that adults with high levels of COVID-19-related suffering use less personal meaning strategies, which, in turn, lead to reduction in positive experiences of life satisfaction and mental health. In the light of these findings, well-being and mental health services can be provided to adults in the face of adversity. To achieve this, prevention and intervention services can be provided to adults, and these services can be based on existential positive psychology (Wong, 1989, 2010, 2011). Mental health professionals may provide meaning-based experiences to buffer the negative impact of suffering during COVID-19 pandemic with life satisfaction and mental health. For example, a comprehensive meaning-based program should be developed by mental health professionals in order to offer psychological effects of traumatic situations (e.g., COVID-19 suffering) on well-being and mental health of people via telephone, where applicable, or online. This is important to address the psychopathology derived from the adversity and contribute to well-being.

Some limitations should be acknowledged when interpreting the results of this study. First, the convenience sampling method was used, and this may jeopardize the generalization of the current findings. Second, the sample was relatively small, composed of a high variability in age (range = 18–83 years), and low representation of male participants (32%). We consider this as another limitation of this study. Future research should be conducted to account for those factors and their impacts on well-being and mental health of individuals with large samples during the health crisis. Third, as the study used a cross-sectional design, it is difficult to draw a conclusion about causality among the study variables. Fourthly, although most measures of this study were validated well and widely used in previous studies, some bias may still raise from using the self-report methods applied in this study. Moreover, in the present study, we were unable to reach those who were directly exposed to COVID-19 such as those infected with the virus. Therefore, future research should focus on examining the mediating effect of personal meaning in the association between suffering and mental health well-being in those exposed to COVID-19. Considering the correlations between personal meaning and life satisfaction and between suffering and mental health, which are relatively high, factor analytic works may be conducted to elucidate that these are separate factors in future studies. Finally, life satisfaction has a multidimensional nature that includes both affective and cognitive dimensions, and this study only focused on the cognitive dimension of life satisfaction. Future work could include both dimensions of life satisfaction to comprehensively understand the impact of pandemic on well-being. Furthermore, the psychometric properties of the suffering measure were examined with the sample of this study. Therefore, there is a need to investigate the psychometrics of the measure with new samples in future research.

In conclusion, this study confirmed the mediating effect of personal meaning on the association between the COVID-19-related suffering and life satisfaction and mental health. It was also found that life satisfaction mediated the association between the COVID-19-related suffering and mental health as well as personal meaning and mental health. As such, this suggests that the COVID-19-related suffering is associated with poor well-being and mental health with multiple pathways. The findings are fruitful in terms of informing researchers and clinicians on the mechanism underlying the associations between the COVID-19-related suffering and well-being and mental health among adults. Researchers, clinicians, and policymakers need to pay special attention to adults to help them deal with the suffering and mental health problems during the COVID-19 pandemic period.

## Data Availability Statement

The datasets generated during and/or analysed during the current study are available from the corresponding author on reasonable request.

## Ethics Statement

The studies involving human participants were reviewed and approved by Agri Ibrahim Çeçen University, Turkey. The patients/participants provided their written informed consent to participate in this study.

## Author Contributions

GA was the main research coordinator of the study, contributed to all steps of the research process, and ran the analysis, and contributed to writing the manuscript. ML contributed to writing the introduction of the manuscript. MY oversaw the study and contributed to writing the discussion of the manuscript. All authors contributed to the article and approved the submitted version.

## Conflict of Interest

The authors declare that the research was conducted in the absence of any commercial or financial relationships that could be construed as a potential conflict of interest.
