# Supplementary material for: Mediating Effect of Personal Meaning in the Prediction of Life Satisfaction and Mental Health Problems Based on Coronavirus Suffering
Source: Front Psychol. 2021 Mar 16;12:638379. doi: 10.3389/fpsyg.2021.638379 (PMC8008149; doi:10.3389/fpsyg.2021.638379)
Supplement: Supplementary file 1 [file Table_1.DOCX]

Suffering Measure during COVID-19 (SMCOVID-19)

To what extent you are suffering from the following during the COVID-19 pandemic. Please indicate how much you are suffering by circling a number on the 5-point scale, from 1 (not at all), to 5 (great deal) which best describes your experiences during the pandemic.

| 1. The spread of COVID-19. | 1 | 2 | 3 | 4 | 5 |
| --- | --- | --- | --- | --- | --- |
| 2. Poor physical health condition. | 1 | 2 | 3 | 4 | 5 |
| 3. Poor mental health condition. | 1 | 2 | 3 | 4 | 5 |
| 4. Poor personal financial condition. | 1 | 2 | 3 | 4 | 5 |
| 5. Social isolation. | 1 | 2 | 3 | 4 | 5 |
| 6. Disruption of daily routines. | 1 | 2 | 3 | 4 | 5 |
| 7. Disruption of personal goals. | 1 | 2 | 3 | 4 | 5 |
| 8. Interpersonal conflicts. | 1 | 2 | 3 | 4 | 5 |
| 9. Discrimination or oppression. | 1 | 2 | 3 | 4 | 5 |
| 10. Toxic political climate. | 1 | 2 | 3 | 4 | 5 |
